# Neuronavigated Right Orbitofrontal 20 Hz Theta Burst Transcranial Magnetic Stimulation Augmentation for Obsessive–Compulsive Disorder with Comorbid Depression and Anxiety Disorders: An Open-Label Study

**DOI:** 10.3390/brainsci14050483

**Published:** 2024-05-10

**Authors:** William F. Stubbeman, Jennifer Yang, Julianne Converse, Melodi Gencosmanoglu, Daisy Morales Ortega, Jordyn Morris, Andrew Sobocinski, Vicky Li, Gabriella Gunawardane, Yana Edelen, Raya Khairkhah, Jillian Perez

**Affiliations:** Stubbeman Brain Stimulation Institute, Los Angeles, CA 90064, USA

**Keywords:** theta burst stimulation, transcranial magnetic stimulation, right orbitofrontal cortex, neuronavigation, treatment-refractory, obsessive–compulsive disorder, major depressive disorder, general anxiety disorder, panic disorder

## Abstract

Background: Despite the availability of pharmacotherapy and psychotherapy for treating obsessive–compulsive disorder (OCD), alternative approaches need to be explored due to the high likelihood of treatment resistance. Neuronavigated 20 Hz theta burst stimulation (TBS-20 Hz), targeting the bilateral dorsolateral prefrontal cortex (DLPFC) augmented with the right orbitofrontal cortex (ROFC), was tested for treating OCD comorbid with depression and anxiety disorders. Methods: A retrospective chart review was performed on fourteen patients treated for moderate-to-severe OCD in a private outpatient clinic. Twelve patients had comorbid major depressive disorder (MDD), and thirteen patients had either generalized anxiety disorder (GAD) or panic disorder (PD). Patients completed the Y-BOCS-SR, BDI-II, and BAI rating scales weekly, which were used to measure the changes in OCD, depression, and anxiety symptoms, respectively. Results: Neuronavigated TBS-20 Hz was sequentially applied to the right DLPFC (RDLPFC), left DLPFC (LDLPFC), and ROFC. A total of 64% (9/14) of patients achieved remission from OCD (Y-BOCS-SR ≤ 14) in an average of 6.1 weeks of treatment (SD = 4.0). A total of 58% (7/12) of patients remitted from MDD (BDI < 13) in an average of 4.1 weeks (SD = 2.8), and 62% (8/13) of patients remitted from GAD/PD (BAI < 8) in an average of 4.3 weeks (SD = 2.5). Conclusions: The neuronavigated TBS-20 Hz sequential stimulation of RDLPFC and LDLPFC, followed by ROFC, significantly reduced OCD, MDD, and GAD/PD symptoms. Randomized sham controls are warranted to validate these results.

## 1. Introduction

Obsessive–compulsive disorder (OCD) is a prevalent psychiatric disorder that affects approximately 2% of the global population [1,2]. Although pharmacotherapy and psychotherapy treatments are available, more than 60% of OCD patients experience a high relapse rate [3]. Moreover, comorbidities with major depressive disorder (MDD), generalized anxiety disorder (GAD), and panic disorder (PD) are relatively common, requiring a comprehensive approach to address these concurrent symptoms related to OCD [4].

In both children and adult patients with OCD, functional imaging studies have observed the hyperactivity in the orbitofrontal cortex (OFC), which corresponds to Brodmann area 47, as part of a cortico-basal ganglia-thalamocortical (CTSC) network activation pattern [5]. A recent study using magnetic resonance imaging (MRI) has also revealed structural abnormalities in the right OFC (ROFC) in patients with drug-naïve OCD, suggesting its contribution to the CTSC loop hyperactivity and ultimately pathogenesis of the obsessive–compulsive symptoms [6]. Furthermore, 1 Hz inhibitory TMS applied to ROFC resulted in a focal decrease in metabolic activity, which correlated with a reduction in the Y-BOCS score [7]. Similarly, significant improvements were observed in OCD comorbid with MDD from targeting the ROFC in addition to the left dorsolateral prefrontal cortex (DLPFC) and bilateral supplementary motor area (SMA) [8]. Based on these studies, we hypothesized that inhibitory TMS targeting ROFC would disrupt the repetitive loop characteristic of OCD, resulting in a concomitant reduction of OCD symptoms.

Our group has previously reported the effectiveness of neuronavigated 20 Hz theta burst stimulation (TBS-20 Hz), specifically targeting the bilateral DLPFC, for treatment-resistant MDD [9]. As theta burst stimulation (TBS) allows for both excitatory and inhibitory stimulation targeting various areas of the brain, a similar observed effect was expected with targeting ROFC, an integral part of the OCD pathology. Moreover, preliminary studies with neuronavigated TBS-50 Hz and TBS-30 Hz have shown promise in reducing OCD symptoms, but TBS-20 Hz has not been tested for OCD [10,11]. Based on the findings in recent publications, we further optimized the TBS-20 Hz protocol by adding ROFC augmentation. We then hypothesized that neuronavigated TBS-20 Hz over a bilateral DLPFC protocol augmented with an inhibitory stimulation of ROFC would effectively lessen obsessive–compulsive symptoms, as well as depression, and anxiety symptoms.

Thus, this study aimed to measure the efficacy of TBS-20 Hz targeting ROFC in addition to bilateral DLPFC for treating OCD patients with comorbid depression and/or anxiety. 

## 2. Materials and Methods

### 2.1. Demographics

IRB-approved retrospective chart reviews (Protocol Number: 520170201) were conducted for fourteen adult patients with a diagnosis of moderate-to-severe OCD (YBOCS-SR ≥ 20) who had completed a minimum of 20 treatments at our clinic. Among these patients, twelve were comorbid with MDD (BDI ≥ 20), and thirteen were comorbid with either GAD or PD (BAI ≥ 15). The moderate-to-severe diagnoses were needed to exclude individuals with mild symptoms, as we deemed their improvement from mild to remission as lacking clinical significance. Other comorbidities included bipolar disorder (*n* = 2), attention deficit hyperactivity disorder (*n* = 1), body dysmorphic disorder (*n* = 1), and anorexia nervosa (*n* = 1). The cohort consisted of six females and eight males, with an average age of 26.4 years (SD = 8.14). All patients were treatment-resistant, as 100% (14/14) of psychiatric medication trials and 82% (9/11) of attempted psychotherapy failed to bring them into remission. The average number of failed psychiatric medications was 4.6 (SD = 2.2), and the duration of current psychiatric episodes was 2.7 years (SD = 1.5). Oral and written informed consent for anonymous research data collection were collected before all treatments.

### 2.2. Targeting and Neuronavigation

Individual 3 Tesla T1-weighted Multiplanar Reconstructed MRI scans were obtained and inputted into the ANT-Neuro Visor 2.0 infrared tracking frameless stereotactic navigation system (ANT-Neuro, Amsterdam, The Netherlands). The MRI scans included 1 mm slices along all three axes, which were used to create a three-dimensional head model of the scalp and brain. RDLPFC, LDLPFC, and ROFC were targeted according to Talairach coordinates: right Brodmann Area 46 (44, 40, 25), its left-side homolog (−44, 40, 25), and Brodmann Area 47 (32, 31, −8), respectively. These Talairach coordinates were previously reported to be effective and safe TMS sites for depression and OCD [12,13]. To ensure optimal field strength, the magnetic field vector was moved to the peak of the nearest gyrus with a perpendicular angle to the local gyrus orientation [14]. The neuronavigation system was active, and the magnetic field was kept within 2 mm of the target for the entirety of the treatment.

### 2.3. Motor Threshold

A MagPro X-100s with a Mag Option and liquid-cooled B-65 figure-eight coil (MagVenture, Farum, Denmark) was used to measure motor thresholds (MT) and administer TMS treatments. The bilateral resting MTs were assessed weekly by visually observing index finger motor movement following the stimulation of the contralateral motor cortex area. Additional MT assessments were performed if any changes occurred in sleep patterns, medication dosage, and caffeine consumption.

### 2.4. Treatment

For the first part of each treatment, 3600–4800 inhibitory pulses were applied over RDLPFC as continuous TBS (cTBS). Subsequently, 4950 excitatory pulses were administered over LDLPFC as intermittent TBS (iTBS) at 2 s on and 8 s off for 27 min. Lastly, 1800–2700 inhibitory pulses were delivered over ROFC as cTBS for two to three minutes [9]. For ROFC stimulation, fewer pulse numbers than the standard 3000 pulses per sessions were used to ensure safety due to its proximity to the eye [15]. All pulses were delivered as TBS-20 Hz which consists of triplet bursts of 20 Hz pulse frequency following 5 Hz inter-burst frequency [9]. The applied magnetic intensities ranged from 90% to 95% of individual MT on the corresponding side [16]. Patients were instructed to wear earplugs during the stimulation of RDLPFC and ROFC and to listen to uplifting subject-selected music during the stimulation of LDLPFC. This paired sensory stimulation was provided to minimize the clicking sound of the equipment used in the treatment, while having minimal executive functional brain activation to avoid activities in the frontal lobe [17,18]. The treatments were administered five days a week until remission was achieved, if not intervened by financial constraints. Y-BOCS-SR, BDI-II, and BAI ratings were completed weekly to evaluate treatment progress [19,20,21,22,23,24]. Remissions for each scale were defined as Y-BOCS-SR ≤ 14, BDI-II < 13, and BAI < 8 [25,26,27]. At the end of each treatment course, remitted patients underwent taper phases, where the number of treatments gradually decreased for the following weeks. Benzodiazepine, muscle relaxants, and gabapentin use were minimized, and alcohol consumption was prohibited during treatment.

### 2.5. Statistical Analysis

Significant differences between pre- and post-treatment measurements were assessed using the Wilcoxon matched-pairs signed-rank test (two-tailed, *p* < 0.05) due to the small sample size (*n* ≤ 14). *p*-values less than 0.05 were considered significant, and all statistical analyses were performed using R Studio ((Version 1.2.5033; RStudio, Inc., Boston, MA, USA) and GraphPad Prism (Version 8.4.3; GraphPad Software, San Diego, CA, USA)).

## 3. Results

**Figure 1:**
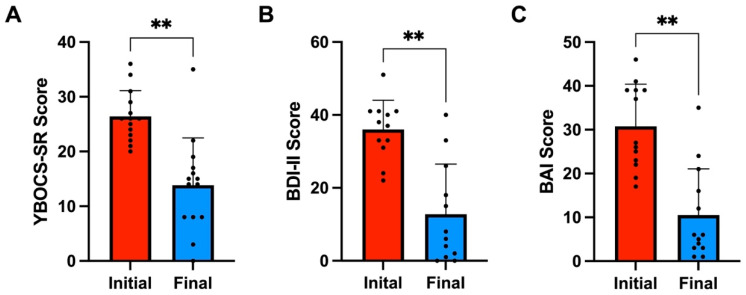
(**A**) Average initial (Red) and final (Blue) YBOCS-SR scale scores for all OCD patients (*n* = 14). (**B**) Average initial (Red) and final (Blue) BDI-II scale scores for all MDD patients (*n* = 12). (**C**) Average initial (Red) and final (Blue) BAI scale scores for all GAD/PD patients (*n* = 13). ** denotes *p*-value less than 0.01.

**Figure 2:**
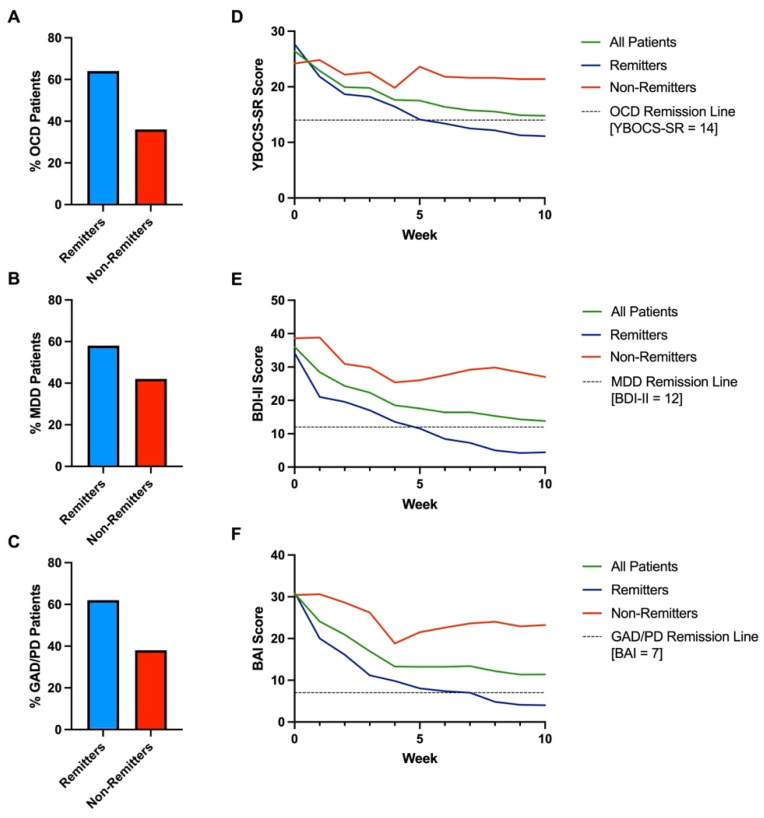
(**A**) Percentage of remitted (Blue) and non-remitted (Red) OCD patients. (**B**) Percentage of remitted (Blue) and non-remitted (Red) MDD patients. (**C**) Percentage of remitted (Blue) and non-remitted (Red) GAD/PD patients. (**D**) Average YBOCS-SR scales over ten treatment weeks for all (Green), remitted (Blue), and non-remitted (Red) patients. (**E**) Average BDI-II scales over ten treatment weeks for all (Green), remitted (Blue), and non-remitted (Red) patients. (**F**) Average BAI scales over ten treatment weeks for all (Green), remitted (Blue), and non-remitted (Red) patients. Five treatment days are defined as one treatment week, rather than seven calendar days. For individual scale score data, please see Figure S1.

A total of 64% (9/14) of patients remitted from OCD (YBOCS-SR ≤ 14) at an average of 6.1 weeks of treatment (SD = 4.0). All patients had an average 48% decrease in Y-BOCS-SR score from 26.4 (SD = 4.7) to 13.8 (SD = 8.6) (Wilcoxon signed-rank test, Z = 3.08, *p* = 0.002). Remitters had a 65% score decrease from 27.7 (SD = 4.8) to 9.7 (SD = 5.7) (Z = 2.67, *p* = 0.004). A large effect size was observed in all patients (Cohen’s d = −1.8) and remitters (Cohen’s d = −3.4). The pre- and post-treatment Y-BOCS-SR score difference for non-remitters was not significant. See Figure 1A and Figure 2A,D.

Moreover, 58% (7/12) of patients remitted from MDD (BDI < 13) in an average of 4.1 weeks of treatment (SD = 2.8). All patients exhibited an average 65% decrease in BDI-II score from 36 (SD = 8.0) to 12.8 (SD = 13.8) (Z = 2.37, *p* = 0.0016). Remitters had a 91% decrease in BDI-II from 34.1 (SD = 8.2) to 3 (SD = 3.1) (Z = 2.37, *p* = 0.002). A large effect size was observed in all patients (Cohen’s d = −1.7) and remitters (Cohen’s d = −5.0). The pre- and post- treatment BDI-II score difference for non-remitters was not significant. See Figure 1B and Figure 2B,E.

Lastly, 62% (8/13) of patients achieved remission from GAD/PD (BAI < 8) in an average of 4.3 weeks of treatment (SD = 2.5). The mean overall BAI score decrease for all patients was 66%, decreasing from 30.8 (SD = 9.6) to 10.5 (SD = 10.5) (Z = 3.17, *p* = 0.002). The average BAI scores for remitters decreased by 88% from 31 (SD = 9.6) to 3.6 (SD = 2.0) (Z = 2.52, *p* = 0.01). A large effect size was observed in all patients (Cohen’s d = −1.9) and remitters (Cohen’s d = −3.6). The pre- and post-treatment BDI-II score difference for non-remitters was not significant. See Figure 1C and Figure 2C,F.

There were no significant baseline differences in age, gender, medication failures, comorbidities, treatment adherences, initial BDI, BAI, and Y-BOCS scores between the remitters and non-remitters for OCD, MDD, and GAD/PD. 

## 4. Discussion

Our results demonstrate the real-world applicability and safety of TBS-20 Hz targeting bilateral DLPFC with ROFC augmentation to treat OCD patients with comorbid depression and anxiety. Similar to the findings of Tadayonnejad et al., significant improvements in OCD and MDD symptoms were observed from a TMS protocol with the inhibitory stimulation of ROFC [8]. However, not only did we see a reduction in symptom severity, but also more than half of our cohort remitted completely from OCD and MDD, as well as GAD/PD. These discrepancies in our outcome may be due to the use of a unique pulse pattern, TBS-20 Hz, rather than the standard 10 Hz repetitive TMS (rTMS), as well as targeting RDLPFC instead of SMA, in addition to LDLPFC and ROFC.

Furthermore, Nauczyciel et al. reported similar findings on the stimulation of ROFC resulting in a significant decrease in Y-BOCS scores for patients with OCD, which correlated with the bilateral decrease in the metabolism in OFC observed using positron emission tomography (PET) [7]. Although Nauczyciel et al. employed different treatment parameters than those used in our study, such as rTMS, double-cone coil, pulse numbers, and stimulation intensity, and no additional targets other than ROFC, we postulate that our TBS-20 Hz protocol alleviates the OCD symptoms in a similar manner, which is by decreasing the metabolic activity of ROFC. The functional hyperactivity of the CTSC loop, which includes OFC, the anterior cingulate cortex (ACC), the basal ganglia, and the thalamus, is widely accepted as the key neurobiological model of OCD [28]. As suggested in a meta-analysis by Gargano et al., TMS targeting OFC may modulate hyperactivity in the CTSC region, counteracting the dysregulation of the related neural circuits and ultimately alleviating OCD symptoms [29].

For the first-line pharmacotherapeutic treatments of OCD, SSRIs and clomipramine are commonly prescribed for the management of obsessive–compulsive symptoms. Although these medications often result in a response, which is typically defined as a 25–35% reduction in YBOCS score from the pre-treatment score, most patients fail to achieve remission [30]. As our cohort consists of treatment-refractory OCD patients with a history of failed medication trials, this study demonstrates the potential of TBS-20 Hz as an effective augmenting agent for non-responders.

Deep Brain Stimulation (DBS) is another form of neurostimulation that is FDA-approved for treating OCD [31]. In patients with treatment-refractory OCD, DBS has also shown significant symptom reduction, achieving an average reduction of approximately 50% in YBOCS scale scores and a 60% response rate. However, DBS requires invasive surgery to implant an electrode in the brain, which manipulates the electrical activity of neurons [32]. Thus, compared to DBS, the preliminary data from our TBS-20 Hz protocol demonstrated an equivalent or superior efficacy with fewer risks and complications, offering a safe and non-invasive alternative [33,34,35].

Despite our promising results, several limitations may affect the interpretation and generalizability of our findings. Due to the small sample size, the statistical power of our analysis is limited with a higher risk for sampling bias. Additionally, the lack of randomized sham controls introduces potential confounding factors, such as the concomitant treatments and placebo effects, which prevent this study from establishing any causal relationship. Despite these limitations, however, the significant clinical improvements observed in this preliminary study will provide a supportive foundation for subsequent randomized sham-controlled studies. Thus, for prospective studies, a larger sample size and sham-controlled randomization are needed to draw cause-and-effect conclusions and enhance the generalizability of research outcomes to a broader population. The long-term sustainability and durability of the observed effects also need to be assessed. If the results observed in this pilot study can be reproduced in future studies, it will provide a significant advancement in the clinical field of OCD.

Although no adverse side effects were observed in our clinic, a few instances of ophthalmological side effects from rTMS targeting DLPFC were reported, such as subconjunctival hemorrhage, posterior vitreous detachment, and retinal detachment [36,37,38]. Due to the anatomical proximity of ROFC to the eyes, it is recommended to use conservative pulse numbers, lower magnetic field intensities, and vectors oriented away from the retina and potentially consult with ophthalmologists in case of pre-existing eye conditions. Increase in eye floaters, change in vision, and any other visual field symptoms were closely monitored during the treatment to prevent any serious ocular side effects. Another serious TMS-related side effect is an increased risk for seizure, which requires the close monitoring of patient’s medical history, alcohol consumption, and possible sleep deprivation [39]. Patients were informed of these potential side effects and gave consent before proceeding with the treatment course.

Additionally, in our clinical practice, we adhere to stringent safety protocols and thus do not initiate TMS therapy for individuals with contraindications for TMS. These may include a history of seizure, the presence of metallic implants above the shoulder, excluding those inside the mouth, and active psychosis [17].

## 5. Conclusions

Substantial improvements in OCD symptoms were observed with neuronavigated TBS-20 Hz sequentially targeting RDLPFC, LDLPFC, and ROFC. A simultaneous alleviation of MDD and GAD/PD symptoms was also observed in patients treated with the proposed protocol. Randomized and sham-controlled trials are needed to validate these promising results.

## Data Availability

The data supporting the findings of this study are available on request from the corresponding author, William F. Stubbeman due to patient privacy reasons.

## Supplementary Materials

The following supporting information can be downloaded at: https://www.mdpi.com/article/10.3390/brainsci14050483/s1, Figure S1. (A) YBOCS-SR scale scores over ten treatment weeks for all patients with moderate-to-severe OCD diagnosis (YBOCS-SR ≥ 20). (B) BDI-II scale scores over ten treatment weeks for all patients with moderate-to-severe MDD diagnosis (BDI ≥ 20). (C) BAI scale scores over ten treatment weeks for all patients with moderate-to-severe GAD/PD diagnosis (BAI ≥ 15). Missing values were addressed using last observation carried forward (LOCF) imputation. Table S1. Demographical and clinical characteristics of the cohort. Table S2. Protocol parameter.
