# Supplementary material for: Neuronavigated Right Orbitofrontal 20 Hz Theta Burst Transcranial Magnetic Stimulation Augmentation for Obsessive–Compulsive Disorder with Comorbid Depression and Anxiety Disorders: An Open-Label Study
Source: Brain Sci. 2024 May 10;14(5):483. doi: 10.3390/brainsci14050483 (PMC11120198; doi:10.3390/brainsci14050483)

Supplementary Materials:

Table S1. Demographical and Clinical Characteristics of the Cohort.

Patients (#)	Average Age (Years)	Gender	Diagnoses (# of Patients)	Average Duration of Illness (Years)	Average Number of Failed Psychiatric Medications (#)	Average Duration of Current OCD Episode (Years)	Patients Taking Psychiatric Medications Concurrently with TMS
14	26.4	6 Female<br>8 Male	Obsessive Compulsive Disorder (14)<br>Major Depressive Disorder (12)<br>Panic Disorder (6)<br>Generalized Anxiety Disorder (7)<br>Body Dysmorphic Disorder (1)<br>Anorexia (1)<br>Attention-Defecit/Hyperactivity Disorder (1)	9.1	4.7	2.7	12

Table S2. Protocol Parameter.

Stimulation Site	Number of Pulses	Motor Threshold	Pulse Frequency (Hz)	Pulses/Burst	Burst Frequency (Hz)	Stimulation Interval (sec)	Intertrain interval (sec)	Uplifting Music	Earplugs
1. RDLPFC	3600-4800	90-95%	20	3	5	240-320	0		x
2. LDLPFC	4950	90-95%	20	3	5	2	8	x	
3. ROFC	1800-2700	90-95%	20	3	5	120-180	0		x

Figure S1. (**A**) YBOCS-SR scale scores over ten treatment weeks for all patients with moderate-to-severe OCD diagnosis (YBOCS-SR ≥ 20). (**B**) BDI-II scale scores over ten treatment weeks for all patients with moderate-to-severe MDD diagnosis (BDI ≥ 20) (**C**) BAI scale scores over ten treatment weeks for all patients with moderate-to-severe GAD/PD diagnosis (BAI ≥ 15). Missing values were addressing using last observation carried forward (LOCF) imputation.

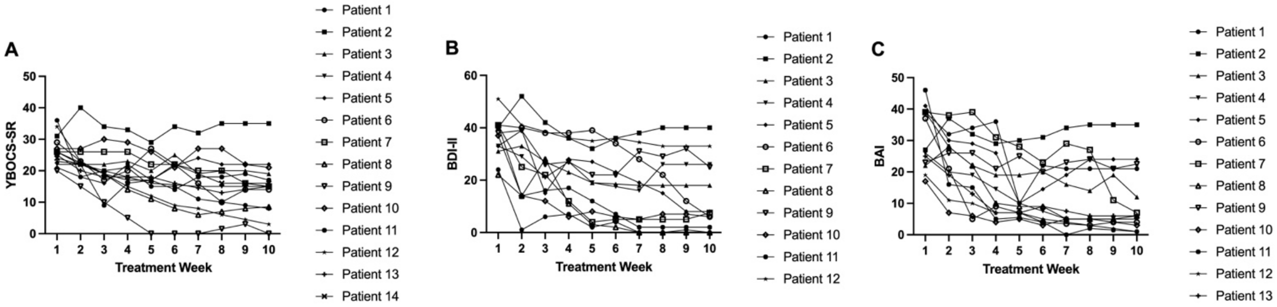

Supplement: Supplementary file 1 [file brainsci-14-00483-s001.zip › brainsci-2931904-SI.pdf]
